# APSIM-based modeling approach to understand sorghum production environments in Mali

**DOI:** 10.1007/s13593-023-00909-5

**Published:** 2024-04-22

**Authors:** Madina Diancoumba, Jana Kholová, Myriam Adam, Mahamoudou Famanta, Benoît Clerget, Pierre C. S. Traore, Eva Weltzien, Michel Vacksmann, Greg McLean, Graeme L. Hammer, Erik J. van Oosterom, Vincent Vadez

**Affiliations:** 1 International Crops Research Institute for the Semiarid Tropics (ICRISAT), BP 320, Bamako, Mali; 2 Leibniz Centre for Agricultural Landscape Research (ZALF), Eberswalder Straße 84, 15374 Müncheberg, Germany; 3 International Crops Research Institute for the Semiarid Tropics (ICRISAT), Patancheru, Andhra Pradesh 502324 India; 4 Department of Information Technologies, Faculty of Economics and Management, Czech University of Life Sciences Prague, Kamýcká 129, Prague, 165 00 Czech Republic; 5 Centre de coopération internationale en recherche agronomique pour le développement (CIRAD), Avenue Agropolis, Cedex 5, 34398 Montpellier, France; 6 Institut Polytechnique Rural de Formation et de Recherche Appliquée (IPR/IFRA), BP 06-Katibougou, Koulikoro, Mali; 7 agCelerant-Senegal, Manobi Africa Group company, Almadies Rue 12 Lot 14, BP 25026, Dakar, Sénégal; 8 International Crops Research Institute for the Semi-Arid Tropics (ICRISAT), Ouakam Rue OKM29 Villa BP 24265, Dakar, Senegal; 9 Agronomy Department, University of Wisconsin - Madison, 371 Moore Hall, Wisconsin, USA; 10 Institut d’Economie Rurale (IER), BP 1813, Bamako, Mali; 11 Queensland Alliance for Agriculture and Food Innovation, University of Queensland, Brisbane, QLD 4072 Australia; 12 French National Research Institute for Sustainable Development (IRD), UMR DIADE, University of Montpellier, 911 Av Agropolis BP65401, 34394 Montpellier, France; 13 LMI LAPSE, CERAAS-ISRA, Thiès, Senegal

**Keywords:** GxE interaction, Crop model, Water deficit scenarios, Photoperiod, CSM335, CSM63E

## Abstract

Sorghum production system in the semi-arid region of Africa is characterized by low yields which are generally attributed to high rainfall variability, poor soil fertility, and biotic factors. Production constraints must be well understood and quantified to design effective sorghum-system improvements. This study uses the state-of-the-art in silico methods and focuses on characterizing the sorghum production regions in Mali for drought occurrence and its effects on sorghum productivity. For this purpose, we adapted the APSIM-sorghum module to reproduce two cultivated photoperiod-sensitive sorghum types across a latitude of major sorghum production regions in Western Africa. We used the simulation outputs to characterize drought stress scenarios. We identified three main drought scenarios: (i) no-stress; (ii) early pre-flowering drought stress; and (iii) drought stress onset around flowering. The frequency of drought stress scenarios experienced by the two sorghum types across rainfall zones and soil types differed. As expected, the early pre-flowering and flowering drought stress occurred more frequently in isohyets < 600 mm, for the photoperiod-sensitive, late-flowering sorghum type. In isohyets above 600 mm, the frequency of drought stress was very low for both cultivars. We quantified the consequences of these drought scenarios on grain and biomass productivity. The yields of the highly-photoperiod-sensitive sorghum type were quite stable across the higher rainfall zones > 600 mm, but was affected by the drought stress in the lower rainfall zones < 600 mm. Comparatively, the less photoperiod-sensitive cultivar had notable yield gain in the driest regions < 600 mm. The results suggest that, at least for the tested crop types, drought stress might not be the major constraint to sorghum production in isohyets > 600 mm. The findings from this study provide the entry point for further quantitative testing of the Genotype × Environment × Management options required to optimize sorghum production in Mali.

**Supplementary Information:**

The online version contains supplementary material available at 10.1007/s13593-023-00909-5.

## Introduction

Sorghum (*Sorghum bicolor* (L.) Moench) is one of the most resilient multi-purpose crops and an important source of staple food for many rural communities in the drier regions of Africa. In Mali, sorghum is the third major staple food crop and along with pearl millet occupies 60% of land cultivated with cereals (FAO 2021). In Mali, sorghum is grown in regions where the rainy season lasts 4 to 5 months (from June to Sept. or Oct.). That period was observed to have decreased since the Sahelian drought of the 1970–1980s, resulting in an observed reduction of the total annual rainfall of ~ 49% compared with that observed in the 1950–1960s (IPCC 2001). The challenging climatic conditions make the sustainable improvement of sorghum production much more difficult. Sorghum breeding programs have made efforts and introduced exotic germplasm resulting in a 2.3% increase in sorghum yield between 1980 and 2013 (Smale et al. 2018). However, despite ongoing efforts, the average yield of sorghum remains low (~ 1 t ha^−1^) compared with its estimated potential 2–3 t ha^−1^ (Fall 2011). Sorghum yield losses are traditionally attributed to the context of crop cultivation; i.e., rain-fed conditions in marginal land with low input management practices (Rattunde et al. 2016)—effects of these production constraints are, however, rarely quantified. Without a quantitative understanding of the production limiting factors, the development and introduction of suitable crop types and management practices that are adapted to this context continue to be difficult (Tardieu et al. 2018; Kholová et al. 2021).

Initial attempts to improve crop adaptability and production across different environmental conditions in the West-African Sub-Saharan sorghum production belt focused on the development of photoperiod (PP) insensitive cultivars (Clerget et al. 2004) as is common in other sorghum improvement programs (Leiser et al. 2012). This approach assumed that eradication of PP-sensitivity would result in cultivars that are short in both height and life cycle and that are more productive because PP-sensitive germplasms are taller (~ 4 m) with a flexible cycle (longer when sown earlier and shorter when sown late) and less productive. However, the developed PP-insensitive cultivars have never been adopted by farmers, because of lower achieved yields than PP-sensitive germplasm. As understood later, because the onset of the rainy season can vary widely, whereas the end of the rainy season is much less variable, PP-responsiveness ensures a narrow window of crop flowering time (in September), irrespective of sowing date, allowing farmers to sow the crop with the onset of the rains (Vaksmann et al. 1996), while also allowing effective usage of temporal water availability in a particular season (Craufurd et al. 1999). Additionally, synchronization of flowering timing could avoid excessive insects and bird damage, grain mold, and an incomplete grain filling for late-maturing genotypes (Summerfield et al. 1991; Vaksmann et al. 1996; Folliard et al. 2004). Optimizing PP responsiveness for Malian sorghum production systems is, therefore, the baseline upon which further adaptation to drought should be built (Craufurd et al. 1999) but, on the other hand, adds one more layer of complexity to any efforts to enhance crop production/resilience in sorghum crop improvement programs (Blum 2005).

Crop modeling approaches have been successfully deployed to explore these complexities in crop improvement programs (Technow et al. 2015). Many studies are being conducted to characterize the production environment of maize, sorghum, rice, and wheat in different parts of the world using crop simulation approaches (Chapman et al. 2000; Heinemann et al. 2007; Chenu et al. 2011, 2013; Chauhan et al. 2013; Kholová et al. 2013; Seyoum et al. 2017). Here, we use Mali as a case study for environment (E) characterization in West and Central Africa (WCA).

Specifically, we aimed to (i) set up the APSIM-Sorghum model for reliable simulations of WCA sorghum production systems; (ii) use the simulation outputs to identify the major drought stress patterns for sorghum growth in Mali and quantify their prevalence across isohyets; (iii) identify the implications for adaptation of PP-sensitive and medium maturing genotypes vs. PP less sensitive and early maturing genotype; and (iv) identify the implications for crop management. The simulation set up is intended to become a guide to breeding efforts toward the design of site-specific and environment-responsive suits of crop-management interventions.

## Materials and methods

### Overview

The APSIM-sorghum module was used to simulate the dynamics of the soil-crop-atmosphere continuum of the sorghum cropping systems in Mali by inputting the essential information on soil, weather, crop type, and crop management practices. The model was parameterized for two soil types with low and high soil water holding capacity (SWHC) that represented the main soil types on which sorghum is widely grown. Daily weather records were available for 18 locations covering 10–35 years, resulting in a total of 459 year-location combinations. Two cultivars, differing in cycle duration and response to photoperiod (PP) were used- CSM63E is a short duration, PP less sensitive material that is predominantly grown by farmers for grain in the Sahelian zone of Mali (isohyets 400–600 mm); whereas CSM335 is a medium maturing PP-sensitive material that is grown for fodder and grain in the Sudanian agro-ecological zone of Mali (600–1000 mm annual rainfall). We simulated, altogether, 1836 crop-soil-year-location combinations. From the daily simulated outputs, we expressed seasonal drought stress scenarios experienced by CSM63E and CSM335 for each combination as a crop water supply/demand (S/D) ratio. Cluster analysis was used to classify the seasonal S/D scenarios into three scenarios and quantify their occurrence across all simulated conditions. The effects of these scenarios on sorghum grain and biomass production across different scenarios were also evaluated.

### Main characteristics of sorghum production regions in Mali

**Fig. 1:**
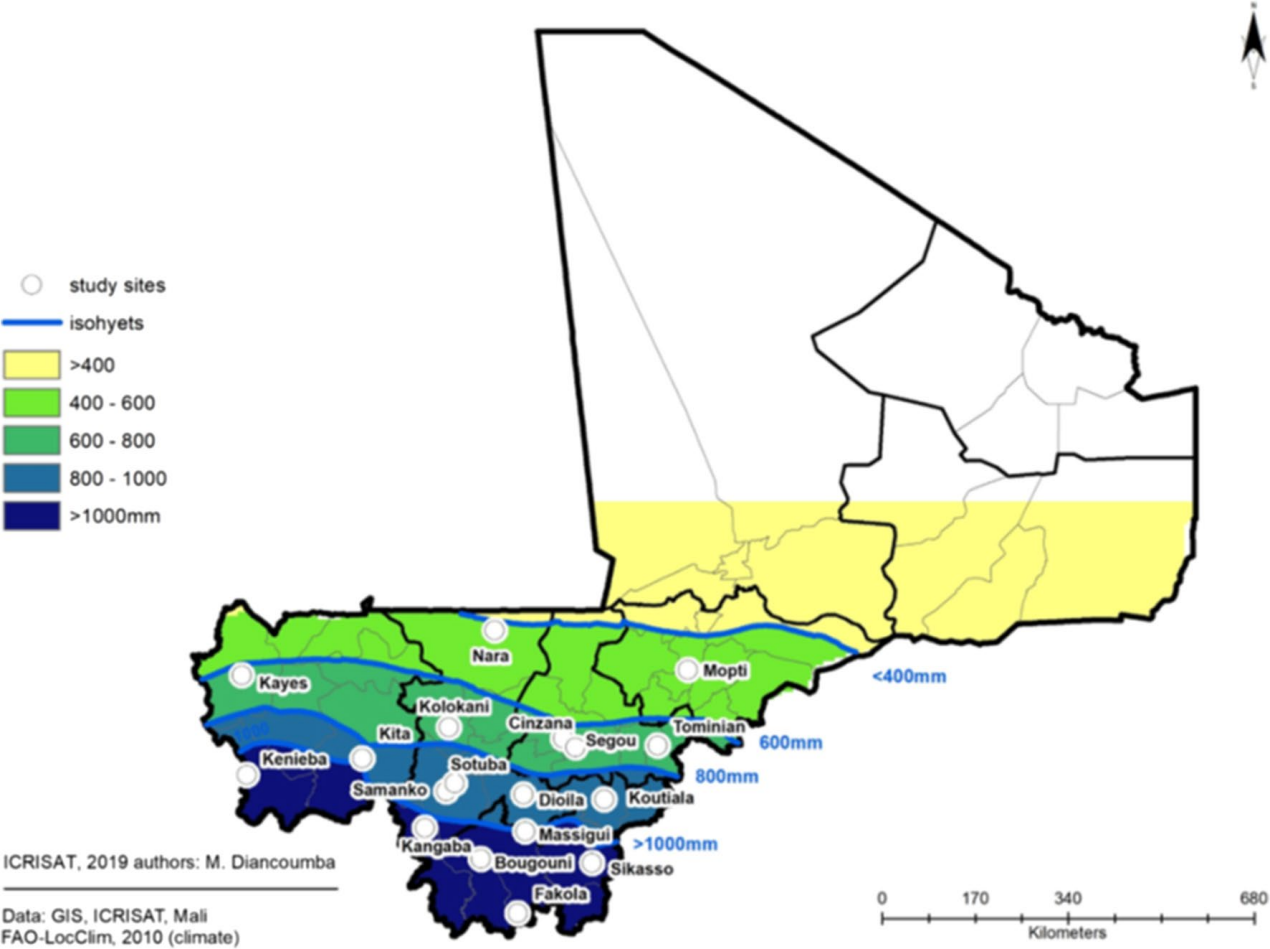
Map of Mali showing the location of the study sites across isohyets (i.e., the blue lines on the map connecting areas of equal rainfall). The Sahelian zone receives < 600 mm rainfall per year, the Sudanian zone receives 600–1000 mm rainfall per year and the pre-Guinean zone receives > 1000 mm rainfall per year.

Malian cropping systems have been previously classified into four major bioclimatic zones which, in principle, follow the variability of rainfall patterns: Sahara (< 200 mm rainfall per year), Sahel (200–600 mm rainfall per year), Sudan (600–1100 mm rainfall per year), and pre-Guinea (> 1100 mm rainfall per year; Soumare 2004). This system has been adapted for generic classification of sorghum production systems which span across the zones with 300–1200 mm rainfall (Smale et al. 2016). The climate across these sorghum production areas is typified by a long dry season followed by a rainy season that lasts from ~ June to October. The duration of the rainy season increases from North to South (Lys 2010), resulting in higher annual rainfall in southern locations (Table 1). The average annual maximum and minimum temperatures typically vary from 30.6 to 37 °C and from 18.6 to 22.7 °C respectively. In our study, we considered four zones based on the aforementioned bioclimatic zonation system and delineated the sorghum growing regions based on isohyets (i.e., contours connecting sites that receive particular amount of rainfall in a given period). Our four zones will be further referred in the text based on the isohyets: isohyets 400–600 mm rainfall per year (part of the Sahelian zone where the climate is arid to semi-arid), 600–800 mm rainfall per year (Northern part of the Sudanian zone where the climate is semi-arid to sub-humid), 800–1000 mm rainfall per year (Southern part of the Sudanian zone where the climate is sub-humid), and isohyets > 1000 mm rainfall per year (most of the pre-Guinean zone where the climate is humid) (Fig. 1). The most severe environments (Sahara and the northern Sahelian zone where the climate is arid) were not considered in this study because sorghum is grown only marginally in that region.

**Table 1:** Description of the 18 locations selected across a North-South gradient of Mali and used in the characterization analysis, providing the isohyets in which the sites are located, the geographical coordinates of each location, start and end of years when weather data were recorded, and the average annual rainfall (Mean rainfall (mm)) observed at each location. No. of years: number of years during which the data were collected.

Bioclimatic zones	Isohyets (mm)	Sites	Long (°W)	Lat (°N)	Start	End	No. of years	Mean Rainfall (mm)
Sahelian	400–600	Nara	7.3	15.2	1980	2003	23	426
		Mopti	4.1	14.5	1980	2005	25	492
Soudanian	600–800	Kayes	11.4	14.4	1980	2004	24	651
		Segou	6.2	13.4	1980	2008	28	661
		Cinzana	5.9	13.3	1980	2010	30	697
		Tominian	4.6	13.3	1980	1990	10	715
		Kolokani	8.0	13.6	1980	2006	26	753
	800–1000	Dioila	6.8	12.5	1980	2008	28	860
		Samanko	8.1	12.5	1980	2014	34	920
		Koutiala	5.5	12.4	1980	2008	58	922
		Sotuba	7.9	12.7	1980	2015	64	942
		Massigui	6.8	11.9	1980	1990	30	943
		Kita	9.5	13.1	1980	2008	70	986
Pre-Guinean	> 1000	Kangaba	8.4	11.9	1980	2006	26	1040
		Fakola	6.9	10.5	1980	1992	12	1054
		Kenieba	11.4	12.8	1980	1997	17	1159
		Bougouni	7.5	11.4	1980	2007	27	1161
		Sikasso	5.7	11.4	1980	2010	30	1170

### Information on the soil-crop-atmospheric continuum required for model input

#### Weather information

Long-term daily weather records (rainfall, maximum and minimum temperatures, and solar radiation) for 18 locations across a North-South gradient of Mali that covered the four bioclimatic zones (Sahelian, North-Sudanian, South-Sudanian, and pre-Guinean zones) were obtained from Mali-Meteo (National Agency of Meteorology in Mali). Records were available for the period 1980–2015, although the number of years varied across sites from 10 to 35 seasons (Table 1). For the sites where only rainfall data were available, the synthetic data AgMERRA (Ruane et al. 2015) was used to fill in the temperature and solar radiation data. As the four zones were defined based on the isohyets (Sahelian: isohyets 400–600 mm rainfall, North-Sudanian: isohyets 600–800 mm, South-Sudanian: isohyets 800–1000 mm rainfall and pre-Guinean: isohyets > 1000 mm rainfall), the simulation outputs were interpreted upon these isohyet zones (Fig. 1).

#### Soil information

To reflect the main soil types on which sorghum is prevalently grown in Mali, two main soil types were parameterized; (1) Plinthaquic Kandustalf (Soil Water Holding Capacity: 62 mm; 101 cm depth) (Gilbert et al. 2003) and (2) Alfisols (SWHC: 156 mm, 120 cm depth) (Clerget et al. Unpublished). Both of these soils are characterized by a low organic carbon content (0.2% for the lower SWHC and 0.37% for the higher SWHC).

#### Crop information: experiments and cultivars

Comprehensive, detailed, and reliable datasets on crop growth and development throughout the season, which are essential for crop model parameterization, are rare or difficult to access for Mali. In this study, the crop data were obtained from experiments conducted by the ICRISAT sorghum program (Clerget et al. 2008) over 8 years at the research centers of ICRISAT, Samanko (12°32 N, 8°04 W) and l’Institut d’Economie Rurale (IER), Cinzana (13°15 N, 5°57 W) in Mali (Table 2). The complete description of the experiments is available in reports provided by ICRISAT sorghum program (available upon request from www.dataverse.icrisat.org). In these files, the detailed records of agronomic practices, inputs, crop agronomic parameters (phenology, grain (kg ha^−1^) and biomass yield (kg ha^−1^) from 8 years observation for 20 cultivars covering the period of 2000–2003 to 2007–2008 were compiled. Additionally, for some experiments and cultivars, we had available data describing plants canopy size over time, including the total leaf number, leaf sizes, and leaf area index. These datasets were used for parameterizing the genetic coefficients of CSM63E and CSM335 into the APSIM-sorghum module.

**Table 2:** Experiment datasets used for traits parameterization and evaluation. The split-plot design was common to all treatments used in the parameterization step while the randomized complete block design was used in the 2013 and 2014 experiments. The phenological phases and the final grain and biomass yield were recorded for all treatments. The datasets used for traits parameterization are available upon request from www.dataverse.icrisat.org.

Genotypes	Steps	Treatments	Sowing year	Measured traits	Sources
CSM63E	Parameterization	Sowing	2000	Height, leaf number appeared, emerged, senesced, dry masses per organ, leaf area	Clerget Reports (2000)
		Validation	2001	Total leaf number	Clerget Reports (2001)
		Sowing densities	2007	Height, leaf number appeared at each growth stage, number of tillers, total leaf number at maturity	Clerget Reports (2007)
		Irrigation	2008	Fresh and dry masses per organ, length from the first node, number of primary branches	Clerget Reports (2008)
CSM335		Sowing	2000	Height, leaf number appeared, emerged, senesced, dry masses per organ, the leaf area	Clerget Reports (2000)
		Sowing	2001	Total leaf number	Clerget Reports (2001)
		Sowing densities	200	Dry masses per organ	Clerget Reports (2003)
		Sowing	2007	Dry masses per organ	Clerget Reports (2007)
		Sowing densities	2008	Dry masses per organ	Clerget Reports (2008)
CSM63E-CSM335	Evaluation	Sowing	2013–2014	Height, leaf number appeared, number of tillers, total leaf number, dry masses per organ, LAI, stem diameter	Akinseye et al. (2017)

Two independent experiments were conducted at ICRISAT, Samanko in 2013 and 2014 and data collected from these experiments were used for model evaluation. The experiment of 2013 had 4 germplasms and 3 sowing dates as treatments while that of 2014 had 2 germplasms, 2 landraces, and 2 sowing dates as treatments and both were laid out as a randomized complete block design with 4 replications. Sowing was done on June 14, on July 9, and on August 5 in 2013; on June 23 and on July 22 in 2014; at 67,000 plants ha^−1^. Details experiments were reported by Akinseye et al. (2017).

These detailed observations focused on two farmer-preferred Guinea-type landraces (CSM63E and CSM335). CSM63E is ~ 210 cm and CSM335 ~ 460 cm tall. Both have open panicles of ~ 35 cm length and an on-farm attainable yield of 1500 kg ha^−1^ (CSM63E) and 1800 kg ha^−1^ (CSM335) with standard agronomic practices (Rattunde et al. 2016). CSM63E is an early-maturing grain sorghum genotype that is less photoperiod-sensitive than other Malian sorghum materials and has been identified to be well adapted to the drier Sahelian region. CSM335, in contrast, is a medium maturing grain sorghum photoperiod sensitive material that is popular in the Sudanian region (isohyets 600–1000 mm).

### Crop model description and functions required to simulate photoperiod sensitive sorghum cultivars

APSIM is a mechanistic crop simulation model enabling reconstruction of the system’s dynamics resulting from the interaction of the soil-crop-atmosphere continuum in a cropping system context (Holzworth et al. 2014). The APSIM-sorghum module has been described in great detail by Hammer et al. (2010, 2016, 2020) and in this study v. 7.10 was used. APSIM has been used to characterize wheat production across environments in Australia (Chapman et al. 2000; Chenu 2015) and sorghum in India (Craufurd and Qi 2010; Kholová et al. 2013; Ronanki et al. 2022) but its use for reliable characterization of African cereals cropping systems has been limited so far.

**Fig. 2:**
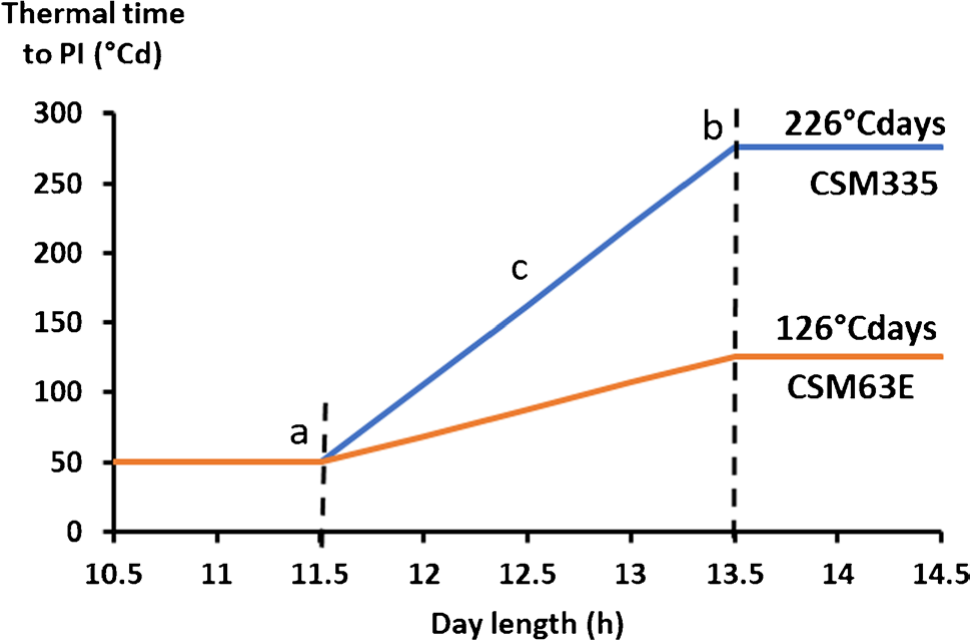
Response of the development rate of genotypes to the length of the photoperiod. A relationship has been established between day length and the thermal time accumulated from the end of juvenile phase to panicle initiation (endjuv_to_init). **a** is the baseline photoperiod, i.e., the minimum day length at which the development rate reaches its minimum (parameter Ppcrit1 in the model); **b** is the maximum day length at which the development rate reaches its maximum (parameter Ppcrit2 in the model); **c** is the photoperiod slope (parameter PP-slope in the model) that characterizes the photoperiod sensitivity of a variety (226°Cd for CSM335 and 126°Cd for CSM63E). PI (Y axis) stands for “panicle initiation.”

The available phenology observations (Table 2) allowed us to derive the coefficients defining the duration of pheno-phases (endjuv_to_init and flower_to_maturity) and coefficients defining the extension of juvenile pheno-phase (endjuv_to_init) responsive to photoperiod (photoperiod_slope: Ppslope) (Table 3), where photoperiod included twilight until the sun is 2.2° below the horizon (Holzworth et al. 2014). Crop response to photoperiod is defined by a triple broken-linear response function (Fig. 2) defined by the minimum (Ppcrit1) and maximum (Ppcrit2) photoperiods, between which increased daily photoperiod decreases the rate of phenological development (extended duration of endjuv_to_init) which is defined as a slope of this linear function (Ppslope; Roberts and Summerfield 1987; Hammer et al. 1993). The default values for Ppcrit1 (11.5 h) and Ppcrit2 (13.5 h) were used in model parameterization in this study and photoperiod_slope was calculated from the linear relationship between thermal time from the end of the juvenile phase to panicle initiation (tt_endjuv_to_init) and the average photoperiod during this developmental phase (van Oosterom et al. 2001; Fig. 2).

**Table 3:** Genotypes specific coefficients obtained from parameterization process using observed data from around 10 agronomic trials. The values with asterisks are variables that have been parameterized. *tt_endjuv_to_init* thermal time from end of juvenile phase to panicle initiation, *tt_flag_to_flower* thermal time from flag leaf appearance to flowering time, *tt_flower_to_start_grain* thermal time from flowering time to start of grain filling, *tt_maturity_to_ripe* thermal time from maturity to ripened grain, *tt_flower_to_maturity* thermal time from flowering to maturity, *dm_per_seed* dry matter per seed, *maxGFRate* maximum growth rate.

Parameters	Units	CSM63E	CSM335
Minimum photoperiod (Ppcrit1)	h	11.5	11.5
maximum photoperiod (Ppcrit2)	h	13.5	13.5
Photoperiod_slope (Ppslope )	°C/h	126*	226*
<tt_endjuv_to_init>	°Cd	50*	50*
<tt_flag_to_flower>	°Cd	170	170
<tt_flower_to_start_grain>	°Cd	80	80
<tt_maturity_to_ripe>	°Cd	1	1
<tt_flower_to_maturity>	°Cd	484*	518*
dm_per_seed	g	0.00083	0.0015*
maxGFRate		0.090	0.045*
Largest leaf multiplier		0.783*	0.698*
Largest Leaf Area Factor (aMaxSlope)		17.163*	32.9*
Intercept for Largest leaf calculation (aMaxIntercept)		− 71.354*	− 296*

Canopy development in APSIM-sorghum (version 7.10) is simulated using the total plant leaf area (TPLA) approach (Hammer et al. 1993). However, this approach could not adequately reproduce canopy development of photoperiod-sensitive genotype with high total main shoot leaf number in response to the extended duration of the period tt_endjuv_to_init. Hence, we incorporated an algorithm based on individual leaf area (ILA) (Birch et al. 1998), which captures the distribution of individual leaf sizes on the main stem using a “bell-shaped” function:

1 $$Y=Y_0\ exp\;\left(a\left(X-X_0\right)^2+b\left(X-X_0\right)^3\right)$$

where *X*_*0*_ is the position of the largest leaf, *Y*_*0*_ the area of the largest leaf, X the position of each individual leaf, “*a*” is an empirical constant determining the breadth of the bell-shaped curve, and “*b*” is an empirical constant determining the skewness of the bell-shaped curve. Each of the parameter’s *X*_*0*_, *Y*_*0*_, *a*, and *b* used for parameterization is a function of the total leaf number. This function, therefore, was defined by 3 crop-specific parameters and allowed modeling of total canopy size depending on the total leaf number developed on the main stem and based on the relationship between the size of the largest leaf and the total leaf number on the main culm:

2 $${Y}_{0}= \mathrm{Max}LNo*a\mathrm{MaxSlope}+a\mathrm{MaxIntercept}$$

where *MaxLNo* is the total leaf number, and *aMaxSlope* and *aMaxIntercept* the slope and intercept for the relationship between the size of the largest leaf and total leaf number. These three crop-specific APSIM parameters in addition to the position of the largest leaf and the *a* and *b* empirical constants define the ILA function.

The genotype-specific parameters regulating the dry matter per seed (dm_per_seed) and the maximum grain growth rate (maxGFRate) were estimated based on the previous study of Tirfessa et al. (2019) on East-African sorghum genotypes.

### Evaluation of cultivar parameters

**Fig. 3:**
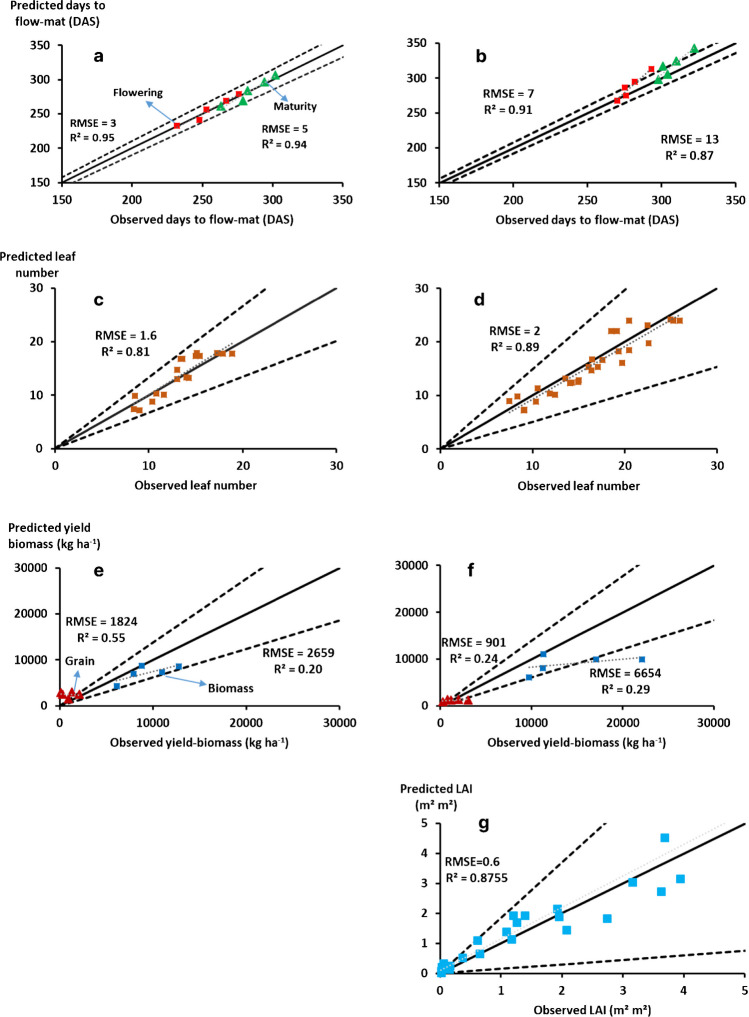
Parameterization of crop-specific coefficients in APSIM-sorghum model. This graph refers to Table 2 (Sowing year 2000, 2001, 2003, 2007, and 2008) and Table 3. The left column is CSM63E while the right column is CSM335. The graph shows the predicted versus observed time (in Days After Sowing: DAS) from **a** Sowing to Flowering and from Sowing to Physiological maturity for CSM63E; **b** the same observations for CSM335; **c** Predicted versus observed total leaf number for CSM63E; **d** the same observations for CSM335; **e** Predicted versus observed (in kg ha^−1^) biomass and grain yield for CSM63E; **f** the same observations for CSM335; Predicted versus observed leaf area indexes were not available for CSM63E; **g** the same observations (m^2^ m^2^) for CSM335. The black lines indicate the 1:1 line and the dashed lines represent the proportion of divergence as per the coefficient of variation (CV) of observed values. The dotted grey lines are the regressions between the predicted and observed values.

For evaluation of the calculated cultivar-specific coefficients (Section 2.4), independent experimental data were used (Table 2). The values of the calculated parameters are shown in Table 3. The evaluation of the goodness of the model fit was done using the correlation analysis between observed and simulated values of phenology, leaf area index (LAI), leaf number, grain, and biomass yield. The criteria for the parameter’s evaluation included the coefficient of determination (R^2^), the mean squared error (MSE), the root mean squared error (RMSE), the normalized root mean square error (NRMSE), the mean absolute error (MAE), and the mean absolute percentage error (MAPE) (Suppl. Table 1) and the proximity to “1:1 line.” Additionally, the space defined by the divergence lines (Fig. 3) was also used as an evaluation criterion. The divergence lines define the space based on the coefficient of variation in the given observations and, according to Soltani and Sinclair (2012), in the relevant model set up ~ 80% of the predicted values should be located within this space. In our case, this criterion was met for almost all the physiological determinants of yield parameterized.

### Identification of the type and frequency of drought stress scenarios and their effects on crop yield

The APSIM-sorghum model was thereafter ran across the 18 locations, with two soils (low and high levels of soil water holding capacity) and two cultivars to characterize the seasonal scenarios of drought stress using the well-established approach reported (Chapman et al. 2000; Chenu et al. 2011; Chauhan et al. 2013; Kholová et al. 2013; Heinemann et al. 2015; Seyoum et al. 2017).

To reflect farming conditions in the study area, the soil moisture in the model was reset to 20% of the fractional available soil water (similar to Chenu et al. 2013; Chauhan and Rachaputi 2014) at the beginning of each growing season. Sowing of sorghum usually occurs around 1 June–15 July and a sowing window was set accordingly, with planting conditioned by an accumulated rainfall of 20 mm within 7 days and stored soil moisture of at least 10 mm. Sowing was forced to start on the last day of the sowing window in case these conditions were not met. All other parameters including plant population (5.7 plants m^−2^) and row spacing (86 cm) were set according to the common farmers’ practices and held constant throughout the simulations. The fertilizer dose was set as per the general recommendation practices across the region of 100 kg ha^−1^ of di-ammonium phosphate at sowing and 50 kg ha^−1^ of urea 45 days after sowing.

The main drought stress scenarios were determined by clustering the drought stress index trajectories simulated across all combinations (crop types, soils, sites, seasons) together using partitioning clustering K-means (Partitioning Around Means - Bhat 2014) and PAM (Partitioning Around Medoids) in R software (R Development Core Team 2011). Separation of individual water availability trajectories based on their similarities into three scenarios allowed for quantification of the scenario occurrences across crop types, soils, sites, and their effects on crop yield. A similar approach has been used by Chenu et al. (2011) and Kholová et al. (2014).

Because the availability of historical weather records varied from site to site, it was necessary to weigh each season (Eq. 3) by the number of seasons available in each particular isohyet zone, in order to compare the scenario occurrences across the entire production region:

3 $$\mathrm{Each}\;\mathrm{season}\;\mathrm{weighing}=\frac{\mathrm{maximum}\;\mathrm{number}\;\mathrm{of}\;\mathrm{seasons}\;}{\mathrm{total}\;\mathrm{number}\;\mathrm{of}\;\mathrm{seasons}\; \;\ast\;\mathrm{number}\;\mathrm{of}\;\mathrm{seasons}\;\mathrm{in}\;\mathrm{given}\; \mathrm{isohyet}}$$

The effects of drought stress scenarios on grain and biomass yield were evaluated within isohyets for all cultivars and soils.

## Results

### Model parameterization and evaluation

In Table 2, we summarized the data, which have been used for the coefficients estimation and the relationship between the simulated and the observed data obtained by using these optimized APSIM parameters. The analysis pointed out the duration of the vegetative phase would be similar for both cultivars in the shortest day–length (i.e., endjuv_to_init ~50°Cd for CSM335 and CSM63E). However, in longer photoperiod, CSM335 took longer to flower (Pp-slope of 126°Cd h^−1^ and 226°Cd h^−1^ for CSM63E and CSM335 respectively), as it is more PP-sensitive. The model reliably captured these cultivars. The optimized duration between the flowering time and physiological maturity within the same datasets was 484°Cd for CSM63E and 518°Cd for CSM335.

Table 3 shows the optimized values of the three crop-specific APSIM parameters defining the ILA function (i.e., Largest leaf multiplier, Largest Leaf Area Factor (aMaxSlope) and Intercept for Largest leaf calculation (aMaxIntercept), Eq. (2)). To acquire these, the detailed observations from experiments 2000/2003 for CSM335 and 2007/2008 for CSM63E were used to derive total leaf number and LAI parameters. The example of the observed and predicted canopy growth dynamics during the season for both genotypes was also shown in the Suppl. Fig. 1.

The parameterization of phenology (RMSE: ~ 4% for both and MAE: 2% and 3% respectively for the flowering time of CSM63E and CSM335) and canopy-related coefficients (Suppl. Fig. 1) resulted in reasonable estimates of biomass (e.g., RMSE: 2% and 1% respectively for the leaf number of CSM63E and CSM335) and grain yield (R^2^, Fig. 3) for CSM63E and CSM335, respectively.

The APSIM-sorghum module set up was tested for its ability to predict the duration of main phenological phases, canopy development, and total biomass and yield of CSM63E and CSM335 using two independent field trials conducted in 2013 and 2014 at ICRISAT Samanko, Mali (Akinseye et al. 2017).

To evaluate the goodness of the fit, we reported the proportion of data lying within the divergence lines (calculated based on the CV of each observed variable as suggested in Soltani and Sinclair 2012) in Suppl. Fig. 2. We found that the phenological phases of both genotypes (CSM63E, CSM335) were predicted reasonably well (RMSE: 4% and 7%—MAE: 3% and 7% respectively for the flowering time of CSM63E and CSM335; Suppl. Fig. 2 a and b) with the majority of the predicted flowering dates located within very narrow CV (~ 4%). For the total leaf number (100%), all points were located within ± 33% and ± 49% respectively for CSM63E and CSM335 (Suppl. Fig. 2c and d). The biomass of CSM63E was predicted relatively well (RMSE: 2659% and MAE: 2139%) with all the data points within ± 38% divergence lines; the biomass of CSM335 was under-predicted by the model (Suppl. Fig. 2e and f). At the same time, the grain yield of CSM63E (and to some extent CSM335) tended to be over-predicted by the model compared with the observed grain yield which was unusually low (e.g., 131 kg ha^−1^ for CSM63E) (Suppl. Fig. 2e and f). This low grain yield observed may be due to factors that were not accounted for by the model but could also be the results of observation inaccuracies. The LAI ~ 80% of the points were within ± 85% for both genotypes (Suppl. Fig. 2g and h).

### Environment characterization

#### Major environment (E) types, their characteristics, and occurrence across cultivars

**Fig. 4:**
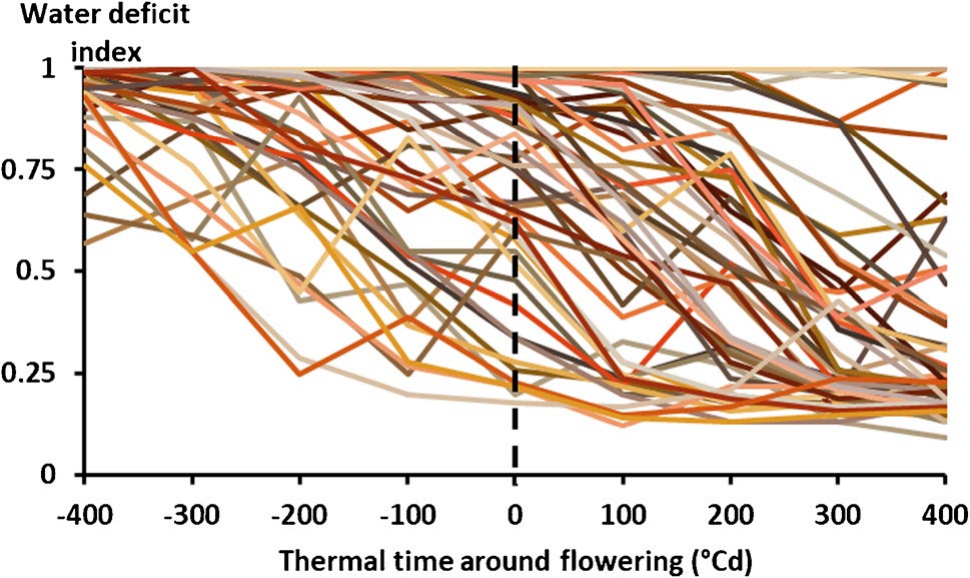
Different lines are all drought stress scenarios identified across soils, crops, years and locations. These are time course trajectories of the crop water status index (the crop water supply/demand (S/D) ratio) through the crop cycle for each year simulated at 18 sites with 2 soils and 2 genotypes. On the Y axis: values of the S/D ratio equal to 1 indicate the crop experiences no stress, whereas values below 1 indicate some stress. On the X axis: the water status index (S/D ratio) was averaged over all 100°Cd intervals from 400°Cd before flowering (− 400, − 300, − 200, and − 100) to 400°Cd after flowering (400, 300, 200, and 100). The dashed vertical line represents the flowering time which is our reference.

**Fig. 5:**
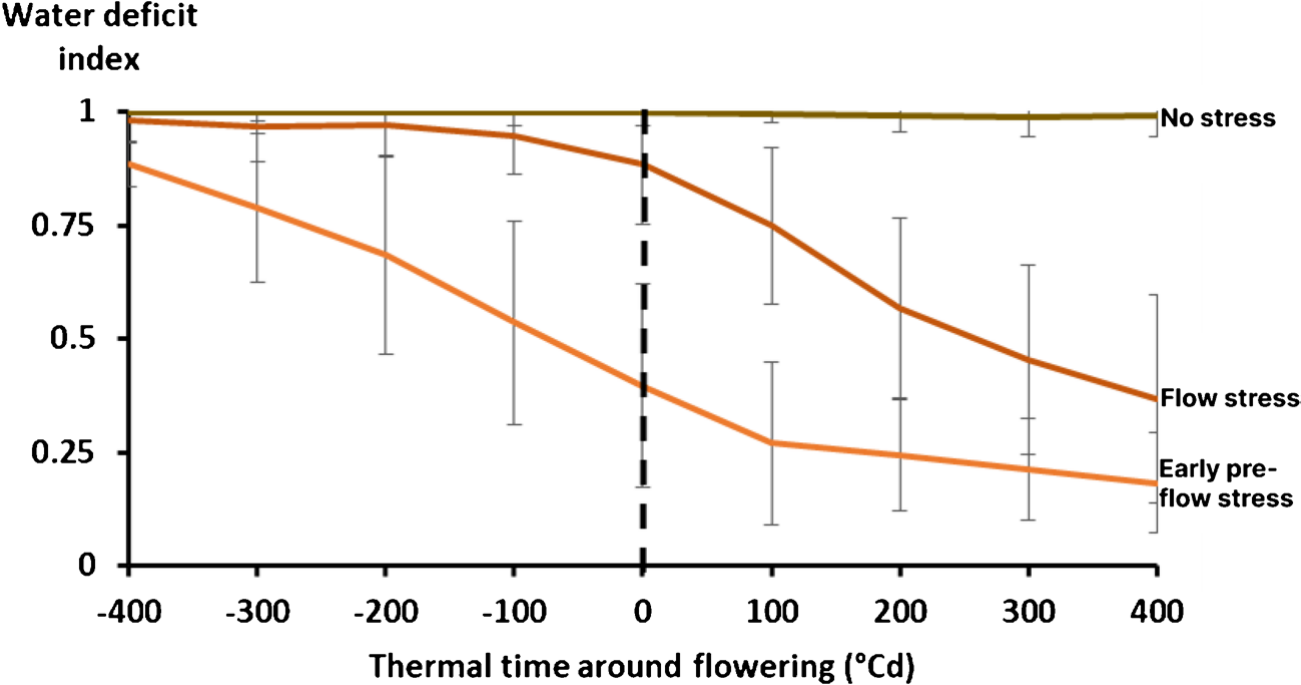
The 3 curves indicate the 3 major drought stress scenarios (no drought stress, early pre-flowering drought stress and flowering drought stress) identified across years, locations and soils for CSM63E and CSM335. On the Y axis: values of the crop water status index (crop water supply/demand (S/D) ratio) equal to 1 indicate the crop experiences no stress whereas values below 1 indicate some stress. On the X axis: the water status index was averaged over all 100°Cd intervals from 400°Cd before and after flowering (± 400°Cd around flowering). The dashed vertical line represents the flowering time that is the reference. The error bars represent the standard deviation of the datasets.

As expected, computation of the supply/demand (S/D) index at all 100°Cd intervals from 400°Cd before to 400°Cd after flowering revealed a large range of variation in water availability simulated across the season-site-crop-soil combinations (Fig. 4). The consequent clustering of all these water availability scenarios revealed three main E types: no stress and two types of end-of-season drought stress that differed in onset of occurrence: before or around flowering (Fig. 5). The no stress had S/D ~ 1 across whole evaluated interval (± 400°Cd around flowering) where the crops did not experience drought stress. The second E type presented conditions where drought stress started before flowering and was not recovered until the end of the season; therefore, was further called an “early pre-flowering drought stress.” The third E type was called a “flowering drought stress” scenario because its onset coincided with the flowering time and was not relieved until physiological maturity for both crop types.

**Fig. 6:**
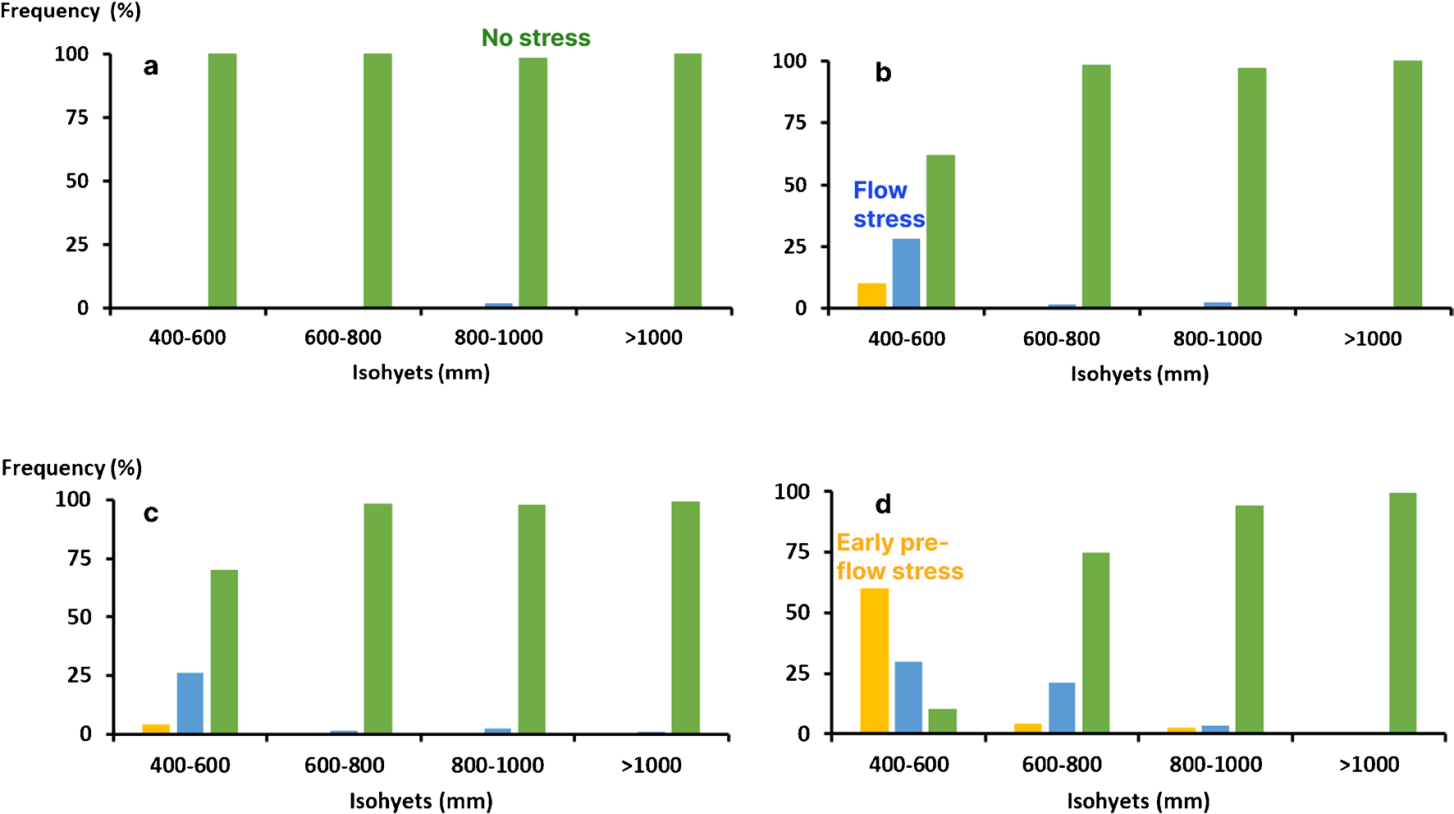
Occurrence of the 3 stress scenarios on higher (**a** and **b**) and lower (**c** and **d**) soil water holding capacity (SWHC), for CSM63E (**a** and **c**) and CSM335 (**b** and **d**) across isohyets (400–600 mm, 600–800 mm, 800–1000 mm and > 1000 mm). The different bars indicate the incidence of the no stress scenario (No stress), the flowering stress scenario (Flow stress), and the early pre-flowering stress scenario (Early pre-flow stress).

Across all conditions, the dominant stress scenario for CSM63E and CSM335 was no-stress with a frequency of occurrence of 95% and 79%, respectively. Moreover, the incidence of drought stress increased more rapidly with CSM335 than CSM63E (Fig. 6). This was associated with a slower rate of development and a greater LAI (Fig. 3), which increased pre-anthesis water demand and hence reduced water availability later in the season.

#### Major environment type’s occurrence across isohyets and soil types

For isohyets > 1000 mm and higher soil water holding capacity (SWHC), there was no incidence of drought stress for either genotype. This indicated that starting soil water and in-season rainfall were sufficient to meet the demand of both genotypes (Fig. 6). As rainfall declined, the incidence of drought stress increased across isohyets. This incidence was greater in soils with LSWHC than HSWHC, as lower SWHC made crops more susceptible to drought stress (Table 4).

**Table 4:** Incidence of the 3 stress scenarios and grain and biomass yield (kg ha^−1^) of CSM63E and CSM335 genotypes used in this study.

Genotypes	Stress scenarios	Incidence of stress scenarios	Grain yield	Biomass yield
CSM335	No-stress	79	1605.3	9543.7
	Flow stress	11	1469.5	8842.2
	Early pre-flow stress	10	913.3	6392.9
CSM63E	No-stress	95	1706.6	4820.6
	Flow stress	4	1392.4	4416.9
	Early pre-flow stress	1	758.6	2812.0

### Genotype × environment interactions

The combined effects of SWHC and genotype influenced occurrence of drought stress patterns with declining rainfall across isohyets. For instance, the early flowering genotype under HSWHC never encountered drought stress (Fig. 6a), even if rainfall ranged 400–600 mm (Sahelian zone). In contrast, late flowering genotype CSM335 encountered drought stress in nearly 50% of the seasons in that zone (Fig. 6b), even though the incidence of drought stress was rare in the isohyet 600–800 mm rainfall.

In soils with low SWHC, the early genotype CSM63E only experienced significant risk of drought stress in the isohyet 400–600 mm rainfall (Fig. 6c), when approximately 40% of the seasons experienced drought stress, in general starting around flowering. In contrast, the late flowering genotype CSM335 (Fig. 6d) already experienced drought stress in nearly 25% of the seasons in the isohyet 600–800 mm rainfall, most of which started around flowering, whereas in the isohyet 400–600 mm rainfall, this genotype experienced end-of-season drought stress in nearly 90% of the seasons, with pre-flowering onset of drought stress most prevalent (Fig. 6).

### Effects of environment on biomass and grain yield

**Fig. 7:**
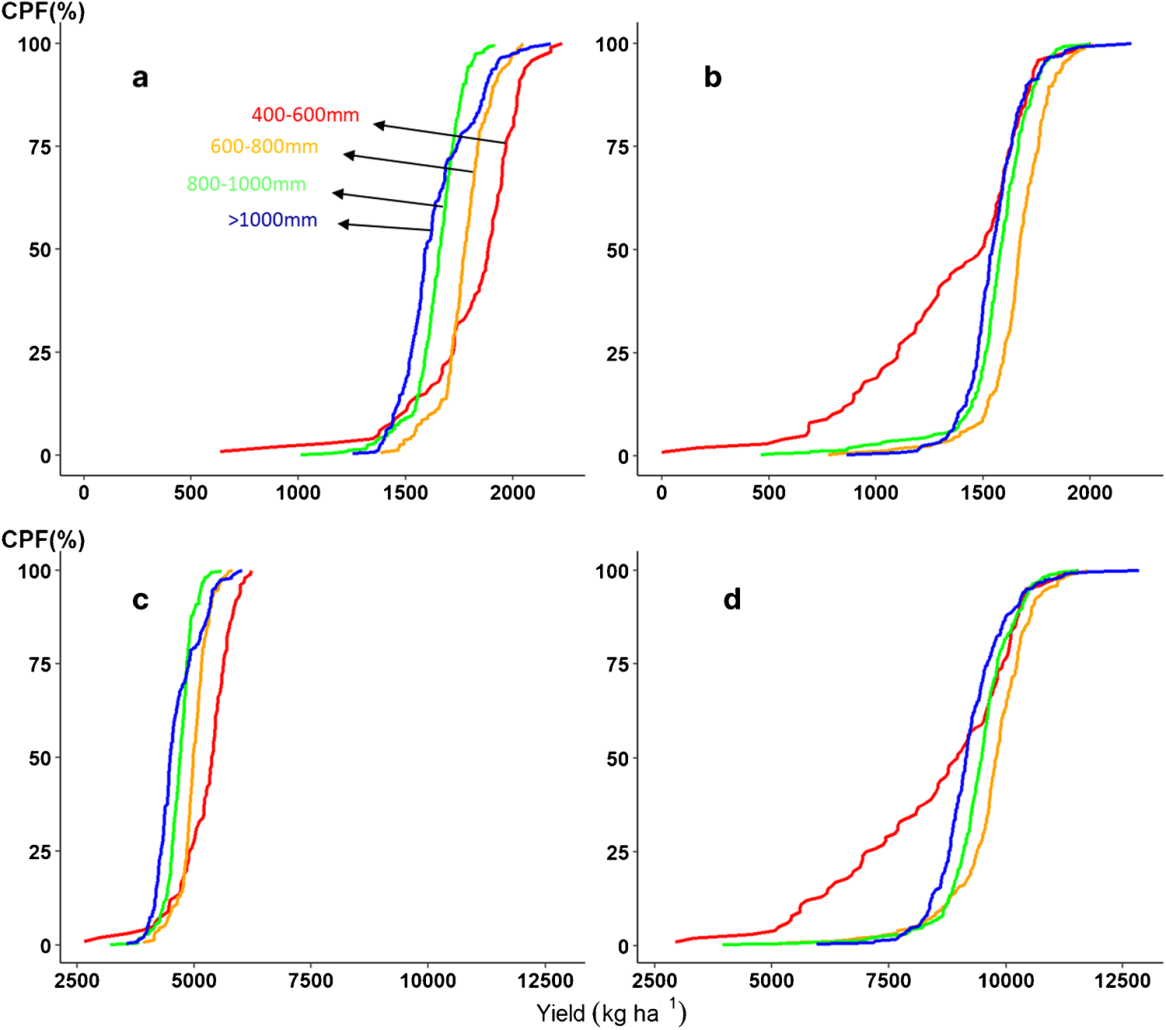
Cumulative probability for grain and biomass yield of CSM63E (**a** and **c**) and CSM335 (**b** and **d**), respectively for each isohyet (400–600 mm, 600–800 mm, 800–1000 mm, > 1000 mm). The Y axis indicates the cumulative probability (CPF in %). The X axis indicates the grain and biomass yield in kg ha^−1^.

Figure 7 shows the cumulative probability distribution of grain and biomass yield simulated for each isohyet over 459 years × 2 soil types × 2 cultivars × 18 sites. The graphs 7a and c show that the grain production varied more for CSM63E across isohyets but the biomass production was comparatively more stable. At the same time, the grain and biomass yield were quite stables for the cultivar CSM335 (Fig. 7b and d) across all the isohyets except of the drought-prone isohyet (400–600 mm rainfall). Both cultivars produced similar average grain yield (1697 kg ha^−1^ CSM663, 1560 kg ha^−1^ CSM335) while the biomass production of CSM335 was higher compared with CSM63E (~ 5000 kg ha^−1^ CSM663, ~ 10,000 kg ha^−1^ CSM335).

**Fig. 8:**
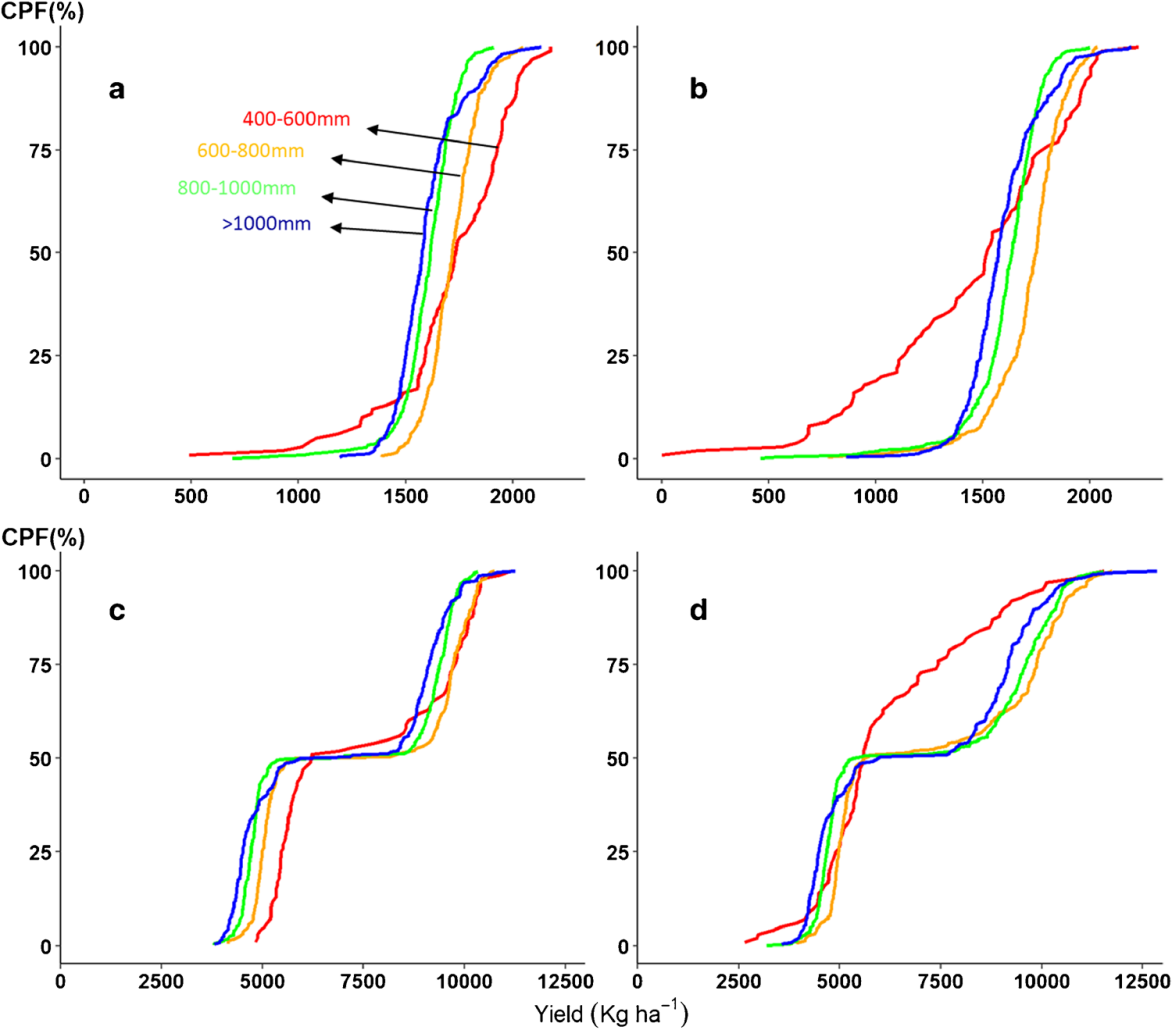
Cumulative probability for grain and biomass yield of both crops (CSM63E and CSM335) simulated on higher (**a** and **c**) and lower (**b** and **d**) soil water holding capacity (SWHC), respectively for each of the 4 isohyets (400–600 mm, 600–800 mm, 800–1000 mm, > 1000mm). The Y axis indicates the cumulative probability function (CPF in %). The X axis indicates the grain and biomass yield in kg ha^−1^.

Figure 8 shows the differences in production between the tested soil types considering both crop types together. Here, we could identify similar trend—the fluctuations in grain and biomass production were more prevalent within the drought-prone isohyet 400–600 mm rainfall (Sahelian zone). Additionally, these fluctuations further enhanced on the shallow soil. Here, we put into evidence that the fluctuations in biomass production were rather specific to the crop cultivation on shallow soil, lower SWHC (Fig. 8b and d). [Note: The expanded width of the distribution in the biomass production (Fig. 8c and d) was caused by accounting for two cultivars with different total biomass production].

## Discussion

### APSIM-sorghum now has an ability to mechanistically simulate photoperiod sensitive cultivars

As compared with other parts of the world, APSIM has not been extensively used in West and Central Africa and particularly in Mali. This could be, at least in part, due to the unique type of crops which are specific with its adaptation to these production regions—i.e., photoperiodism. The photoperiod sensitivity of the sorghum crop allows the crops to be sown during the wide planting window while maturing at the narrow time window (details in Vaksmann et al. 1996). The need for simulating the photoperiod sensitive crops was fully realized under the Agricultural Model Inter-comparison and Improvement Project (AgMIP) (MacCarthy et al. 2018; Adam et al. 2020; Akinseye et al. 2020). These studies used the released version of APSIM and attempted to reflect the photoperiod sensitivity but did not use the mechanistic algorithm loop to simulate this particular crop feature. Our study implemented a mechanistic loop into the APSIM v.7.10 which linked the changes in canopy size to the variable duration of vegetative phenological phases (adopted from Birch et al. 1998). This allowed the model to respond to the changes in photoperiod by variable canopy size (Carberry et al. 1993). We envisage that improving crop model ability to capture the variation between photoperiod-sensitive cultivars (vegetative stage, flowering time, and canopy) will help, e.g., plant breeders to evaluate the performance of potential cultivars in multiple environments and across spatiotemporal resolutions (Folliard et al. 2004; Stöckle and Kemanian 2020). This ability allows the model for use in “interpretative” applications.

### Drought stress occurs less often than expected

Our results show that the frequencies of drought stress were much less prevalent across the tested region than expected (Fig. 6). Drought stress mostly affected the photoperiod-sensitive genotype CSM335 in the isohyet 400–600 mm rainfall (Fig. 6b and d) and, up to certain extend the photoperiod-less sensitive CSM63E in same isohyet (Fig. 6a and c). The main type of drought stress pattern was the terminal drought stress, with variable timing of the onset across isohyets and soil types (i.e., starting before or around crop flowering). This is consistent with observation that rainy season in WCA is very unpredictable (Vaksmann et al. 1996; Folliard et al. 2004; Soumare et al. 2008; Akinseye et al. 2015). Drought stress occurrence across isohyets and soil types was frequently the result of the combination of the highly variable distribution of rainfall, the lower amount of moisture present in the soil, and the timing of sowing.

### Genotype x environment interactions

Our study pin-pointed and quantified the advantages and risks associated with cultivation of relatively more photoperiod-sensitive (CSM335) and less photoperiod-sensitive (CSM63E) crops in Mali. The main difference between these 2 crop types were apparent in the production potential and production stability within each isohyet and this coincided with the occurrence of drought stress in northern isohyets (< 800 mm rainfall). The grain and biomass production of the medium maturing photoperiod-sensitive genotype, CSM335, when compared with CSM63E, was more stable (Fig. 7c and d) across all isohyets except of isohyets < 600 mm rainfall. In the isohyets > 600 mm rainfall, CSM335 produced similar amount of grain and substantially more biomass compared with CSM63E, thus, appeared to be a more stable cultivar choice for these regions. Conversely, in isohyets < 600 mm rainfall, the production of the cultivar CSM335 was frequently devastated by early pre-flowering stress (nearly every other season) while CSM63E experienced the similar type of stress only once in 50 years. CSM63E might be a less risky option for these latitudes (< 600 mm rainfall). These simulations outputs fit our experimental data well and agree with the prior reports documenting CSM335 having a longer duration and higher biomass accumulation compared with CSM63E (Adam et al. 2018). The capacity to mechanistically simulate the photoperiod sensitivity of sorghum crop with its consequences on crop agronomy empowered us to design the crop “blueprint” as per the requirements of the target stakeholders (e.g., breeding programs).

### Relevance of the developed modeling set up for sorghum crop improvement and sorghum production stabilization in Mali

In Mali, resources are extremely limited and the environments targeted by the crop improvement programs are immensely complex. The increasing levels of environmental degradation and climatic risks further undermine the agricultural sector and call for novel approaches to understand and address these escalating issues (FAO 2017). In this situation, we envision the APSIM modeling set up developed in this work could expand the spatiotemporal scales of the crop testing system and would complement the traditional multi-location crop evaluation approach practiced by local breeding programs. Furthermore, the presented modeling framework could assist the breeding programs to pre-test the effect of cropping system interventions and would provide the required foresight to develop site-specific and balanced agri-system resource utilization strategy.

In this particular work, we adapted APSIM to begin understanding the system heterogeneity linked to the water availability across the sorghum production zones of Mali. At this stage of the framework development, our work suggested that some of the high-rainfall regions (specifically isohyet > 1000 mm) in Mali might be under-exploited and we aim to use the developed APSIM modeling setup to further evaluate the potential of intensified cultivation practices (e.g., increased fertilizer input, higher plant population or different plant type). Conversely, northern regions with much lower rainfall (i.e., isohyet < 600 mm) and severe water limitations during the season might benefit from conservation agricultural practices (mulching, shorter crop duration, etc.). Our study supports the generally accepted concept; i.e., the need for the development of geographically specific crop-management production packages (Cooper et al. 2021) and here we provide the tool to design such production strategy in silico.

### Possible limitations of the current study and on-going efforts

Despite the implementation of the ILA-based algorithms to capture canopy development of photoperiod-sensitive material, these, in the current form, could not reliably reproduce the canopy growth dynamics of the long-cycle sorghum material (such as IS15401 that is a tall highly photoperiod-sensitive type, adapted to the Guinean zone). The efforts are underway to collect more experimental data to support the future development of the reliable algorithms to reflect these crop types.

In addition, it is important to note that the APSIM setup presented did not capture the response of the crop to other abiotic (waterlogging, genetic variation for crops responsiveness to soil phosphorus) or biotic stresses (pests and diseases) which might also contribute to the yield losses across the Malian sorghum production region.

The present model setup is subject to continuous improvement, which will be guided by our increasing knowledge of the West-African sorghum production system and collection of more data. Nevertheless, the current simulation setup should be sufficient to begin testing the genotype × management (G×M) options for distinct geographies. In the next step, we plan to extend the resolution and spatiotemporal targets of simulations with the higher resolution of current and future meteorological information (e.g., NASA-AgMERRA; Ruane et al. 2015, already used in e.g., Ronanki et al. 2022). This would expand our understanding of the system heterogeneity as well as the quantification and understanding of the crop production yield gaps (e.g., Hajjarpoor et al. 2018) at the scales relevant to crop improvement programs.

## Conclusion and way forward

In this work, we succeeded to reliably parametrized two sorghum crop types representing the commonly grown Malian cultivars into the APSIM-sorghum module. The parameterization of this photoperiod-sensitive material became realistic only when the mechanistic algorithms, which connected the changes in total leaf numbers with the crop phenological development, were incorporated into APSIM code. Consequently, we created the APSIM modeling set up to characterize the sorghum production environments in Mali, focusing on the water availability. We were able to detect the drought stress scenarios and, for the first time, quantified their occurrence and their consequences on grain and biomass production. These drought stress scenarios affected sorghum production mainly in the low-rainfall Sahelian production zone (isohyet 400–600 mm rainfall) while in the remaining zones the drought stress did not appear to be a major production limitation for the tested cultivars.

We intend to use hereby developed simulation basis for in silico pre-testing the genotype × management (G×M) interventions effects on crops agronomic performance across Mali and other African locations.

### Supplementary Information

Below is the link to the electronic supplementary material.

Supplementary file1 (DOCX 657 kb)

## Data Availability

The datasets generated and/or analyzed during the current study are available from the corresponding author on reasonable request.
