# Supplementary material for: APSIM-based modeling approach to understand sorghum production environments in Mali
Source: Agron Sustain Dev. 2024 Apr 22;44(3):25. doi: 10.1007/s13593-023-00909-5 (PMC11035133; doi:10.1007/s13593-023-00909-5)
Supplement: Supplementary file 1 — Supplementary file1 (DOCX 657 kb) [file 13593_2023_909_MOESM1_ESM.docx]

**Supplementary material**

**APSIM-based modeling approach to understand sorghum production environments in Mali**

Madina Diancoumba^1,2^, Jana Kholová^3,4*^, Myriam Adam^5^, Mahamoudou Famanta^6^, Benoît Clerget^5^, Pierre C.S. Traore^1,7^, Eva Weltzien^1^, Michel Vacksmann^5,8^, Greg McLean^9^, Graeme L. Hammer^9^, Eric J. van Oosterom^9^, Vincent Vadez^3,10^.

*^1^International Crops Research Institute for the Semiarid Tropics (ICRISAT), BP 320, Bamako, Mali.*

*^2^Leibniz Centre for Agricultural Landscape Research (ZALF), Eberswalder Straße 84, 15374 Müncheberg, Germany*

*^3^International Crops Research Institute for the Semiarid Tropics (ICRISAT), Patancheru 502 324 Andhra Pradesh, India.*

*^4^Department of Information Technologies, Faculty of Economics and Management, Czech University of Life Sciences Prague, Kamýcká 129, Prague, 165 00, Czech Republic.*

*^5^Centre de coopération internationale en recherche agronomique pour le développement (CIRAD), Avenue Agropolis, 34398 Montpellier Cedex 5, France.*

*^6^Institut Polytechnique Rural de Formation et de Recherche Appliquée (IPR/IFRA), BP 06- Katibougou, Koulikoro Mali.*

*^7^Mobile-2-Web business services to generate value chain efficiency everywhere and for everyone (Manobi), Fenêtre Mermoz 25026, Dakar, Sénégal.*

*^8^Institut d’Economie Rurale (IER), BP 1813, Bamako, Mali.*

*^9^University of Queensland, Queensland Alliance for Agriculture and Food Innovation, Brisbane, QLD 4072, Australia.*

*^10^Institut de Recherche pour le Développement (IRD), 911 Avenue Agropolis, 34394 Montpellier, France.*

**Corresponding author, email:* [*J.Kholova@cgiar.org*](mailto:J.Kholova@cgiar.org)

**Supplementary Figures and Table: APSIM-sorghum model parametrization and validation**

The supplementary figure 1 shows the dynamic of canopy growth (leaf number and leaf area index) for CSM63E and CSM335, simulated by APSIM-sorghum model (lines) as compare with the observed data (dots) collected in the field over the growing seasons (refer to Table 2). The canopy growth data used for parameterizing the genetic coefficients of CSM63E and CSM335 into the APSIM-sorghum module was not equality available for both genotypes in same growing seasons (e.g., leaf number was available for CSM63E in 2007 while it was not for CSM335). Overall, leaf number was accurately predicted by the model while the leaf area index was sometimes over predicted. This could be attributed to the observed data collected because measuring the leaf area index is challenging rather than the crop model used, as APSIM has been extensively evaluated including in African context under optimum and water limited conditions (Seyoum et al. 2017; MacCarthy et al. 2018; Adam et al. 2020; Akinseye et al. 2020). The metrics (e.g., RMSE and R^2^) used to assess the goodness of fit of the observed versus simulated crop parameters are reported in Suppl. Table 1.


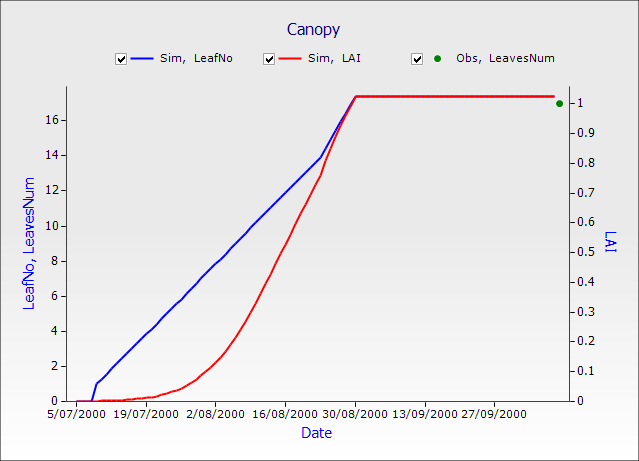

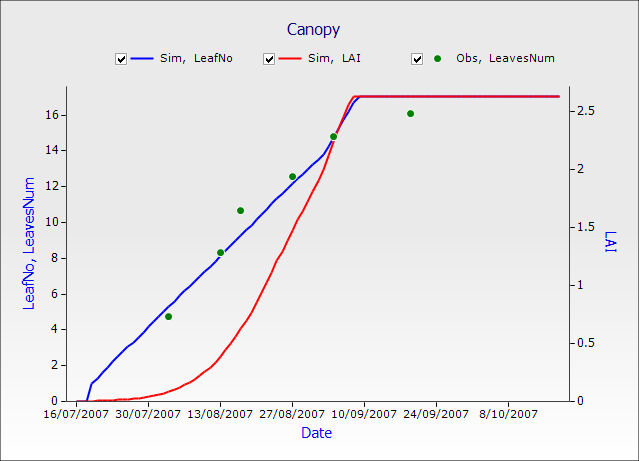

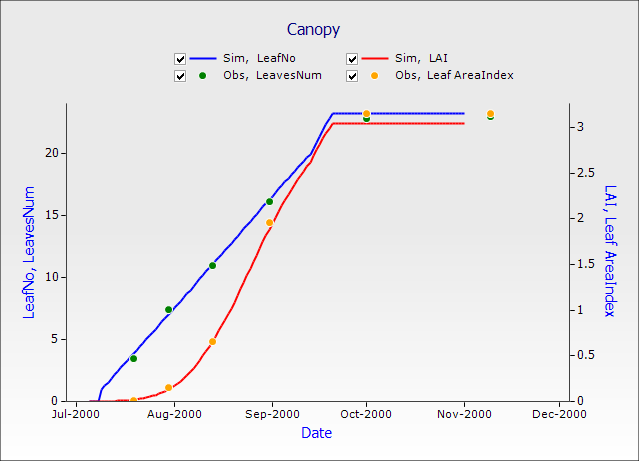

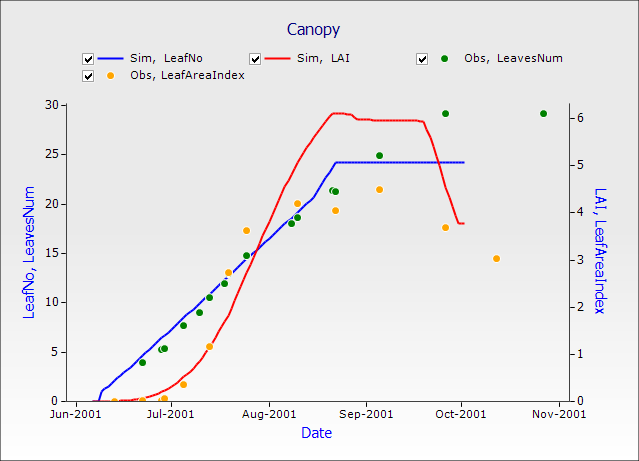

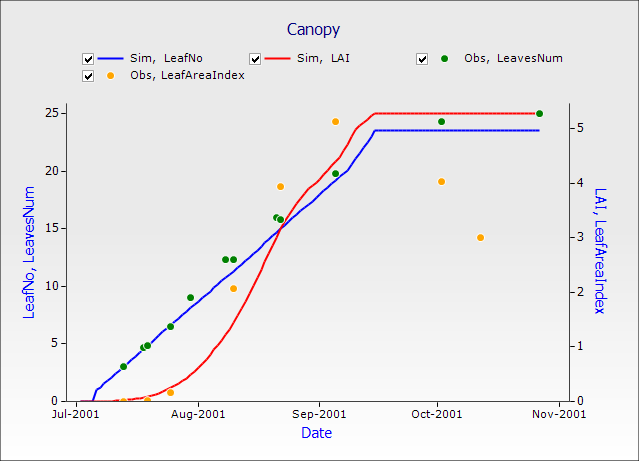

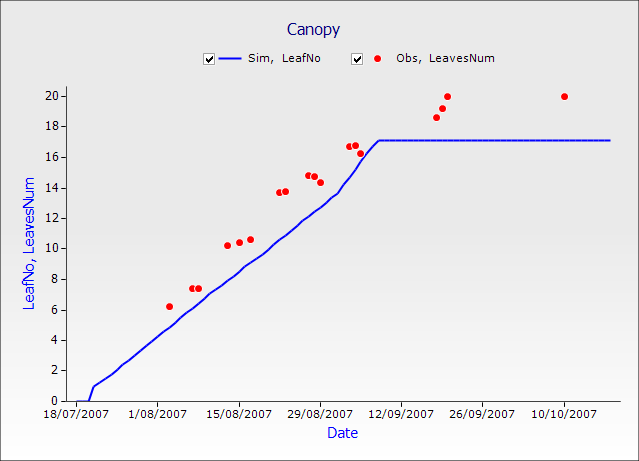


**CSM63E**

**CSM335**

Figure 1. Observed and predicted canopy growth dynamic of both genotypes

Suppl. Figure 2 shows the evaluation results of APSIM-sorghum model performance. Data used for model evaluation were collected in tow independent experiments trials in 2013 and 2014, in ICRISAT Samanko, Mali (Table 2). CSM63E is in the left column while CSM335 is in the right column. The graph shows the observed versus predicted pheno-phases from sowing to flowering and from flowering to physiological maturity (upper row), for the total leaf number (second row from the top) and for the grain yield and final biomass (bottom row). The evaluation of the goodness of the model fit was done using the correlation analysis between observed and simulated values for all the parameters considered. The criteria used to evaluate the calculated cultivar-specific coefficients are available in Suppl. Table 1.


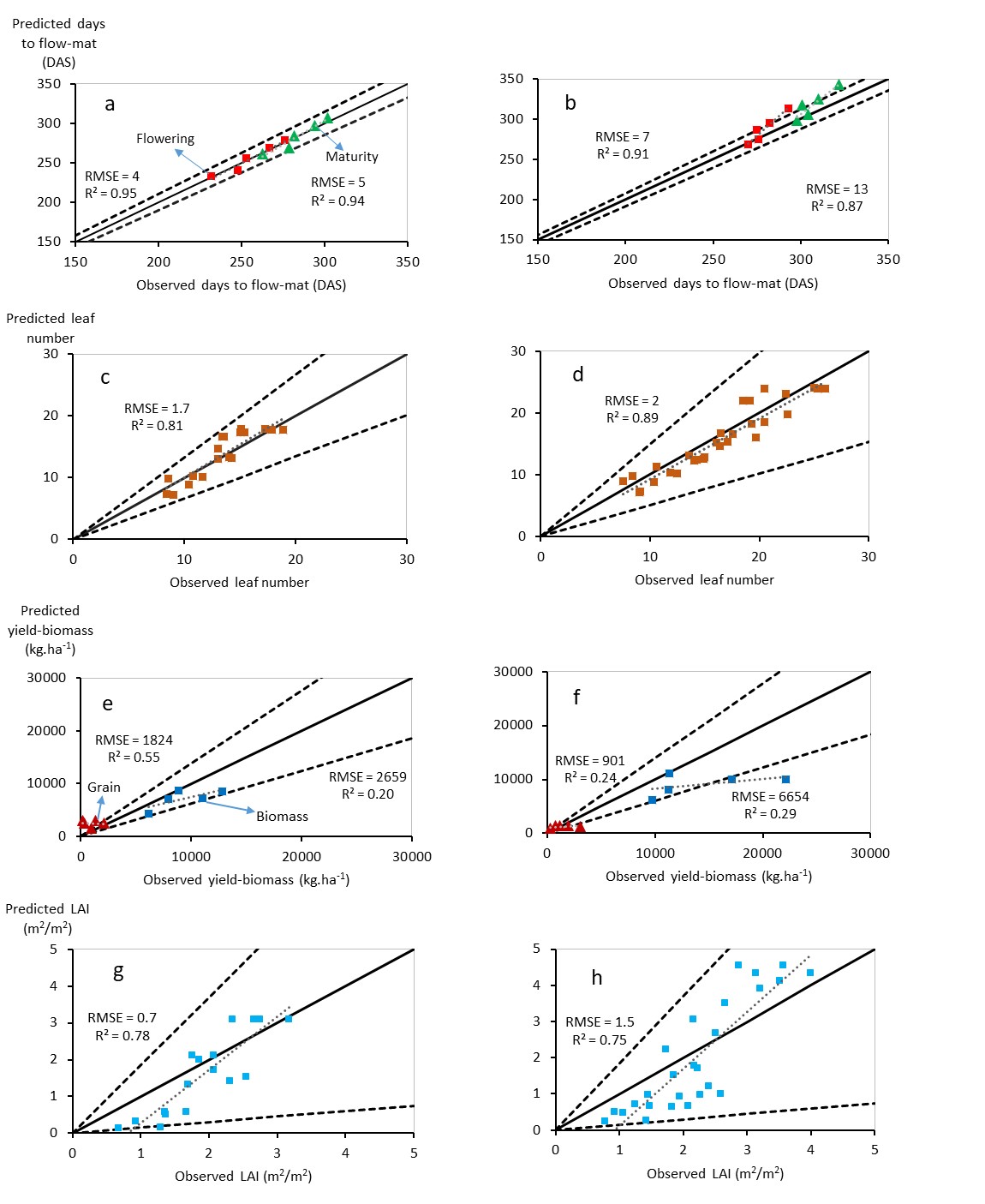


Figure 2. Evaluation of the model performance. This graph refers to Table 2 (Sowing 2013 and 2014) and Table 3. Predicted versus observed time (in Days After Sowing: DAS) between sowing and flowering and between sowing and physiological maturity for CSM63E (a); the same observations for CSM335 (b); Predicted versus observed total leaf number for CSM63E (c); the same observations for CSM335 (d); Predicted versus observed (in kg ha^-1^) biomass and grain yield for CSM63E (e); the same observations for CSM335 (f); Predicted versus observed (in m^-2^/m^-2^) leaf area index for CSM63E (g); the same observations for CSM335 (h). The black lines indicate the 1:1 relationship line. The dashed lines represents the proportion of divergence as per the coefficient of variation (CV) of the observed values. The dotted lines are the regressions between the predicted and observed values.

**Supplementary Table 1.** Criteria used to evaluate the goodness of the model fit that resulted from the correlation analysis between observed and simulated values of the parameters considered in the parametrization and evaluation process. MAE: mean absolute error, MAPE: mean absolute percentage error, RMSE: root mean square error. R^2^: coefficient of determination is also available in Fig. 3 and Suppl. Fig. 2.

| Process | Genotype | Model evaluation criteria | Flowering | Maturity | Yield | Biomass | Leaf number | Leaf Area Index |
| --- | --- | --- | --- | --- | --- | --- | --- | --- |
| Parametrization | CSM63E | MAE | 2.3 | 3.5 | 747.5 | 1558.3 | 1.8 |  |
|  |  | MAPE | 0.0 | 0.0 | 0.3 | 0.2 | 0.1 |  |
|  |  | RMSE | 4.3 | 4.2 | 1113.6 | 2396.5 | 2.1 |  |
|  | CSM335 | MAE | 3.3 | 5.3 | 519.4 | 2503.0 | 0.9 | 0.5 |
|  |  | MAPE | 0.0 | 0.0 | 0.2 | 0.2 | 0.1 | 0.9 |
|  |  | RMSE | 4.0 | 5.8 | 694.6 | 2967.6 | 1.1 | 0.7 |
| Evaluation | CSM63E | MAE | 3.2 | 4.8 | 1565.4 | 2139.0 | 1.5 | 0.6 |
|  |  | MAPE | 0.0 | 0.0 | 6.3 | 0.2 | 1.0 | 0.7 |
|  |  | RMSE | 3.8 | 5.3 | 1824.4 | 2659.1 | 1.7 | 0.7 |
|  | CSM335 | MAE | 7.0 | 10.8 | 736.9 | 5240.4 | 1.8 | 0.9 |
|  |  | MAPE | 0.0 | 0.0 | 0.9 | 0.3 | 0.7 | 0.7 |
|  |  | RMSE | 7.0 | 13.6 | 901.3 | 6654.3 | 2.0 | 1.5 |
